# Comparison Between a Standard and SalivaDirect RNA Extraction Protocol for Molecular Diagnosis of SARS-CoV-2 Using Nasopharyngeal Swab and Saliva Clinical Samples

**DOI:** 10.3389/fbioe.2021.638902

**Published:** 2021-03-29

**Authors:** Sofía N. Rodríguez Flores, Luis Mario Rodríguez-Martínez, Bernardita L. Reyes-Berrones, Nadia A. Fernández-Santos, Elthon J. Sierra-Moncada, Mario A. Rodríguez-Pérez

**Affiliations:** ^1^Instituto Politécnico Nacional, Centro de Biotecnologia Genomica, Reynosa, Mexico; ^2^Laboratorio Estatal de Salud Pública de Tamaulipas, Secretaría de Salud de Tamaulipas, Ciudad Victoria, Mexico; ^3^Universidad Autónoma de Nuevo León, Facultad de Ingeniería Mecánica y Eléctrica, San Nicolás de los Garza, Mexico

**Keywords:** molecular diagnosis, RNA extraction protocol, clinical samples, COVID-19, SARS-CoV-2

## Abstract

During the COVID-19 pandemic, a certified laboratory of Tamaulipas, Mexico has processed over 100,000 samples of COVID-19 suspected patients, working a minimum of 100 tests daily. Thus, it would be beneficial for such certified laboratories nationwide to reduce the time and cost involved in performing the diagnosis of COVID-19, from sample collection, transportation to local lab, processing of samples, and data acquisition. Here, 30 nasopharyngeal swab and saliva samples from the same COVID-19 individuals were assessed by a standard nucleic acid extraction protocol, including protein lysis with proteinase K followed by binding to column, washing, and elution, and by the SalivaDirect protocol based on protein lysis, skipping the other steps to reduce processing time and costs. The genomic RNA was amplified using a SARS-CoV-2 Real-Time PCR kit. A variation (*P* > 0.05) in the 95% CIs = 72.6%–96.7% was noted by using the SalivaDirect protocol and saliva samples (sensitivity of 88.2%) in comparison to those of standard protocol with oropharyngeal swab samples (95% CIs = 97.5%–100%; sensitivity of 100%) as reported elsewhere. However, when using nasopharyngeal swab samples in the SalivaDirect protocol (sensitivity of 93.6%; 95% CIs = 79.2%–99.2%), it was in concordance (*P* < 0.05) with those of the standard one. The logical explanation to this was that two samples with Ct values of 38, and 40 cycles for gene E produced two false negatives in the SalivaDirect protocol in relation to the standard one; thus, there was a reduction of the sensitivity of 6.4% in the overall assay performance.

## Introduction

Rapid and precise detection of COVID-19 is necessary for surveillance and control of the pandemic. qRT-PCR and nasopharyngeal swabs are the recommended test and sample to be used (Azzi et al., 2020). The sensitivity of qRT-PCR depends on the protocol, the sample analyzed, the number of clinical samples, and other factors (Yam et al., 2003). However, the collection of nasopharyngeal or oropharyngeal samples causes discomfort to patients and may cause bleeding, especially in patients with thrombocytopenia (Chan et al., 2020). Collection of samples requires close contact between healthcare workers and patients, representing a risk factor of transmission of the virus because samples collected may contain live viruses (To et al., 2020b). These findings reinforce the use of barrier protection equipment as a control measure for all healthcare workers (Fakheran et al., 2020).

The two main components of a SARS-CoV-2 nucleic acid extraction kit are proteinase K and silica columns. Proteinase K is widely used in scientific research and industries mainly because only few proteins resist to it. It has potent activity to denature proteins, even in the presence of strong detergents, and it can be used to isolate viral genomic material (Yang et al., 2016). Thus, proteinase K is massively used in COVID-19 laboratories. Moreover, RNA purification based on silica columns is used mainly because it allows fast extractions with good yields of high-quality nucleic acids. However, silica columns are expensive, especially for non-developed countries (Nicosia et al., 2010).

Nowadays, qRT-PCR assays have enabled the diagnosis of COVID-19 from saliva (To et al., 2020a,b). On April 14, 2020, the United States-Food and Drug Administration (US-FDA) authorized the first saliva-based nucleic acid test for emergency use in COVID-19 diagnosis (Rutgers, 2020); namely, a saliva test in which the sample can be collected at home and mailed in for testing (Food and Drug Administration [FDA], 2020a). On August 15, 2020, the US-FDA authorized the SalivaDirect protocol, based on reduced times by skipping silica column wash in acid nucleic extraction protocol (Food and Drug Administration [FDA], 2020b; Vogels et al., 2020).

It has been shown than saliva sample collection for diagnosis and viral load monitoring of SARS-CoV-2 has many advantages. It requires less stringent conditions and can be used as a screening tool that highly minimize the chance of exposing healthcare workers in comparison to nasopharyngeal swabs (To et al., 2020a). Therefore, this sample collection can decrease the risk of nosocomial SARS-CoV-2 transmission, and it is not necessary the presence of trained personnel (Fakheran et al., 2020).

Previously, it was demonstrated that saliva has a high concordance rate with nasopharyngeal aspirate in the detection of respiratory viruses, including coronaviruses such as SARS-CoV-2 (To et al., 2019b, 2020a, 2020b; Williams et al., 2020; Zheng et al., 2020). It has been reported that the use of saliva for coronaviruses detection such as SARS-CoV-2 is more sensitive and reliable than the use of nasopharyngeal swabs (To et al., 2017; Chan et al., 2020; Wyllie et al., 2020). Therefore, saliva has been used to screen respiratory viruses on patients without respiratory symptoms (To et al., 2019a).

Because of the ease of sample collection, self-collected saliva has been used in Hong Kong for diagnostic test, at the hospital accident and emergency department, at the airport, and in contact tracing for COVID-19 cases (Hong Kong SAR Government, 2020).

However, we notice that saliva collection had several problems that can be common when doing it daily. Also, we demonstrate the utility of the nucleic acid extraction SalivaDirect protocol for RNA extraction of SARS-CoV-2 and the assessment of nasopharyngeal swabs and saliva clinical samples using a standard protocol and the SalivaDirect protocols.

## Materials and Methods

### Clinical Samples and SARS-CoV-2 Nucleic Acid Extraction Protocols

Thirty nasopharyngeal swab clinical samples were examined using a standard protocol following the manufacturer’s instructions (QIAamp Viral RNA Mini, Qiagen, MD, United States) at a certified laboratory. Briefly, 140 μl of homogeneous (vortexed) nasopharyngeal swab clinical sample with 560 μl of AVL buffer was mixed and incubated at room temperature (RT) for 10 min for lysing proteins. Ethanol (560 μl) was added followed by nucleic acid binding to the column containing the filter and collector tube. It was centrifuged at 6000×*g*, and the liquid was discarded through the filter tube. Then, 750 μl of AW1 buffer was added and drowned by centrifugation followed by the same procedure but using AW2 buffer. A brief spin (1 min) removed the remaining buffers. The nucleic acids were eluted into a sterile 1.5-ml tube by adding 60 μl of elution AVE buffer, incubated at RT for 1 min, centrifuged at 8,000 × *g* for 1 min, and stored at −80°C for further analysis.

The same nasopharyngeal swab clinical samples of the same COVID-19 patients were also examined by using the SalivaDirect nucleic acids extraction protocol (Vogels et al., 2020). The main distinction between both protocols is that in the SalivaDirect protocol, the wash, column binding, and elution steps were skipped. Briefly, 50 μl of the homogeneous (vortexed) nasopharyngeal swab clinical sample was added to 2.5 μl of proteinase K (50 mg/ml) for lysing proteins. To inactivate proteinase K, the homogenate was incubated at 95°C for 5 min and stored at -80°C for further analysis. The SalivaDirect assay was performed using saliva clinical sample from the same COVID-19 individuals.

### SARS-CoV-2 Nucleic Acid Amplification Protocol

The RNA amplification of SARS-CoV-2 was performed following the manufacturer’s instructions of the SARS-CoV-2 Real-Time PCR kit (Vircell, Granada, Spain). Positive and negative controls were tested in parallel to validate the reaction.

A sample was considered positive if the cycle threshold (Ct) value obtained in E gene was less than 40 and the internal control, RdRp gene, showed amplification. A sample was considered negative if the assay did not show amplification for gene E but it did for the RdRp gene. If the assay did not show an amplification signal for the RdRp gene, the acid nucleic extraction protocol was repeated. The acid nucleic amplification assay was done all over again if the Ct value for gene E was over 40. As the gold protocols (from Qiagen and Vircell) used here have a sensitivity of 100% as reported elsewhere (World Health Organization [WHO], 2020), it was used as reference to decide whether or not an individual was truly positive or negative to the tests.

### Statistical Analysis

The 95% exact Clopper-Pearson confidence intervals (CIs) surrounding the point estimate of the SalivaDirect protocol were calculated using the diagnostic test evaluation calculator (MedCalc software^1^; February 17, 2021) in relation to the gold standard protocol with nasopharyngeal swab clinical samples. Estimates for which the 95% CIs did not overlap were considered to be significantly different at the *P* < 0.05 level.

## Results

A variation (*P* > 0.05) was noted when 30 saliva clinical samples were assessed by the SalivaDirect protocol (sensitivity of 88.2%; 95% CI = 72.6%–96.7%) in relation to the gold standard protocol (95% CIs = 97.5%–100%; sensitivity of 100%) as reported elsewhere (World Health Organization [WHO], 2020; Table 1). This was because four out of 30 saliva clinical samples had late Ct values (ranging from 34 to 38 amplification cycles) for gene E, which are associated with low viral loads in the samples. However, the standard protocol with nasopharyngeal clinical samples was able to detect the virus in those samples with Ct values of up to 40 cycles.

**TABLE 1:** Comparison of three protocols for acid nucleic extraction of SARS-CoV-2 (*n* = 30) individuals diagnosed with COVID-19 from nasopharyngeal swab and saliva clinical samples.

**Protocol**	**Gene E**	**Gene Rp**	**No. of positive samples/*n***	**Sensitivity^$^ %**	**95% Confidence intervals^$^ %**
	**Ct-values^&^ cycles**	**Ct-values^&^ cycles**			
Nucleic acid extraction standard protocol with nasopharyngeal exudate^%^ (QIAamp Viral RNA Mini, Qiagen, MD, United States)	16–40	23–34	30/30	100	97.5–100 (World Health Organization [WHO], 2020)
Nucleic acid extraction SalivaDirect protocol with saliva^£^ (Vogels et al., 2020)	9–39	12–40	26/30	88.2	72.6–96.7
Nucleic acid extraction SalivaDirect protocol with nasopharyngeal exudate^£^ (Vogels et al., 2020)	17–38	18–39	28/30	93.7	79.2–99.2

***^%^**Proteinase K of unknown concentration. ^£^ 2.5 μl (50 mg/ml) Proteinase K. ^&^Range. ^$^Calculated using MedCalc Software Ltd (2021).*

The same 30 nasopharyngeal swab clinical samples from same COVID-19 individuals were assessed by the SalivaDirect protocol (Table 1). Only two out of 30 nasopharyngeal swab clinical samples were found to be false negatives (sensitivity of 93.7%; 95% CIs = 79.2%–99.2%), indicating that saliva clinical samples are less sensitive than nasopharyngeal swab samples. Thus, no significant difference (*P* > 0.05) was seen between the two assays using nasopharyngeal swab clinical samples. In addition, eight saliva clinical samples in the standard protocol showed Ct values over 36, and these samples also showed positive results using the SalivaDirect protocol. Interestingly, there was concordance in the positive result using one nasopharyngeal swab sample with a Ct value of 40 for both assays (i.e., standard and SalivaDirect protocols).

## Discussion

Saliva used as diagnostic sample provides an opportunity for simpler and more efficient tools for virus diagnosis, especially during the critical episodes of viral disease outbreaks (Khurshid et al., 2019). Furthermore, literature mentions that the screening times will be reduced by instructing patients to spit into a sterile bottle (Fakheran et al., 2020). However, in our gathering of samples with trained personnel, collection of nasopharyngeal swabs took less than 3 min, but getting saliva lengthened the process due to the wait for the patients to be able to salivate.

On several occasions, the patients produced largely sputum instead of saliva. In other studies, patients were asked to cough out saliva and samples were mainly sputum (Cheng et al., 2020; To et al., 2020b; Zheng et al., 2020). Also, other patients cannot generate saliva.

Variation in viral load between saliva and sputum samples has not yet been reported (Chen L. et al., 2020; Zheng et al., 2020), but it is complicated to manipulate sputum in the laboratory due to its viscosity, in addition to containing higher concentrations of degrading enzymes that can interfere with detection processes.

In the clinical setting, we have observed that during the saliva collection, many patients contaminated the outside of the container with their saliva. This puts staff at risk, or it can lead to cross-sample contamination. Due to this and because saliva may allow transmission of virus (To et al., 2020b), we consider that it is necessary to use personal protection equipment and to follow all the established safety measures as with that of the nasopharyngeal swab collection. Saliva self-collection gives the possibility of collecting samples outside the hospitals, but it could damage the sample during patient handling or transportation. For these reasons, we do not consider viable the option of self-collection outside hospitals.

Here, the saliva samples were taken, stored in a cooler, and transported directly to the laboratory. Usually, the samples are collected, then sent out to the clinic, and later on the samples are sent to the laboratory (the next day if they are in another town). Thus, keeping the sample in a transportation medium should be considered. Lippi et al. (2020) suggested that sample collection errors and transportation factors also have an impact on assay detection accuracy. It has shown that liquid Amies medium, PBS, or maintenance medium can be used for homogenization of saliva samples (Williams et al., 2020; Wong et al., 2020).

To solve the logistic problems associated with sample collection, it is necessary to develop an easily and standardized method to collect the samples as well as for keeping the samples: Namely, putting the saliva sample into a sterile container and then viral transport medium (To et al., 2017), putting the saliva direct into a sterile container with viral transport medium (To et al., 2019a) or without viral transport medium (Moreno et al., 2020).

In the laboratory, the only drawback, as aforementioned, was the difficulty of working with viscous samples. It has been shown that the processing of nasopharyngeal swabs with proteinase K and without silica column washes was as effective as the standard protocol, indicating that this methodology can reduce time and costs. It was also demonstrated that nasopharyngeal swabs should be recommended as the sample to be used (Azzi et al., 2020).

Thirty patients were confirmed to harbor SARS-CoV2 nucleic acids using a standard protocol with nasopharyngeal swab clinical samples. When saliva clinical samples from the same patients were assessed with the SalivaDirect protocol (Vogels et al., 2020), four were found to be false negatives (sensitivity of 88.2%; 95% CI = 72.6%–96.7%). A sensitivity of detection of the virus using gold standard protocols with oropharyngeal clinical samples was documented to be 100% (95% CIs = 97.5%–100%) (World Health Organization [WHO], 2020), indicating a significant variation (*P* > 0.05) in the assays.

The consequence for the decrease of the sensitivity was noted in samples with late Ct values (Ct values = 34–38 cycles) for gene E. In qRT-PCR, Ct scan is suggested as an auxiliary diagnostic method to avoid reporting false results and to increase sensitivity (Li et al., 2020). Chen J.H. et al. (2020) reported significant differences in the median Ct values for nasopharyngeal swabs in comparison to that on saliva samples (26.8 vs 29.7, *p* = 0.0002), indicating that COVID-19 detection assay is less sensitive with saliva samples than nasopharyngeal swabs, which is in concordance with our findings.

Regarding the concordance between detection results of SARS-CoV-2 using nasopharyngeal swabs and saliva clinical samples, different sensitivity rates have been reported (Table 2). Here, the 30 nasopharyngeal swab clinical samples from same COVID-19 patients using the SalivaDirect protocol had a sensitivity of 93.7% (95% CIs = 79.2%–99.2%) which was not different (*P* > 0.05) of that when saliva clinical samples were used (sensitivity of 80.2%; 95% CIs = 72.6%–96.7%). This is consistent as reported elsewhere (Azzi et al., 2020). Also, concordance (*P* > 0.05) in nasopharyngeal swab samples using the SalivaDirect protocol (95% CIs = 79.2%–99.2%) and the gold standard protocol (95% CIs = 97.5%–100%) as reported by World Health Organization [WHO] (2020) was noted (Supplementary Table 1).

**TABLE 2:** Number of positive/individuals examined (percentage) when using either nasopharyngeal swabs or saliva clinical samples assessed by qRT-PCR for SARS-CoV-2.

**Nasopharyngeal swab samples**	**Saliva samples**	**References**
Positive/individuals examined (%)	Positive/individuals examined (%)	
12/12 (100)	11/12 (91.7)	To et al., 2020b*
23/23 (100)	20/23 (87.0)	To et al., 2020a
13/13 (100)	4/13 (30.76)	Chen J.H. et al., 2020
39/39/100)	33/39 (84.0)	Williams et al., 2020
25/25 (100)	25/25 (100)	Azzi et al., 2020
47/65 (72.3)	37/42 (88.0)	Zheng et al., 2020*
55/58 (94.8)	52/58 (89.7)	Chen L. et al., 2020
47/53 (89)	41/53 (77)	Jamal et al., 2020

*Saliva samples including saliva and sputum samples.

Infections by SARS-CoV-2 can be detected at high titers with tests based on saliva samples. It has been reported to be 3.3 × 10^6^ and 10^11^ copies per cm^3^ (Cheng et al., 2020; Pan et al., 2020). In sputum, it has been reported to be 10^9^ and 1.34 × 10^11^ copies per cm^3^ (Pan et al., 2020; To et al., 2020a).

However, an observational cohort showed that viral load in saliva was highest following symptom onset in the first week and then subsequently declined with time (To et al., 2020a). However, in one of these studies, viral RNA could still be detected from a third of patients for 20 days or longer, and in one patient, it was detected 25 days after symptom onset (To et al., 2020a). Wang et al. (2020) reported that the median duration of viral shedding in sputum was 34 days (24–40) and 19 days (14–25) in nasopharyngeal swabs. Similarly, Park et al. (2020) reported that the median duration of SARS-CoV-2 viral detection after hospitalization was 34 days (22–67). After resolution of symptoms, SARS-CoV-2 was detected for a median of 26 days (9–48). For this, unlike nasopharyngeal swabs, saliva only can be used for early detection and subsequent viral load monitoring (To et al., 2020a,b; Wyllie et al., 2020).

Also, consideration should be the detection of SARS-CoV-2 in saliva by rapid antigen-based tests. In Mexico, the Institute for Epidemiological Diagnosis and Reference has approved rapid antigen-based kits from swab samples. The PanbioTM COVID-19 Ag rapid test device and Sofia 2 SARS antigen FIA kit can give results in 15 min; however, they showed a sensitivity of 85 and 80%, respectively, in patients who were in the first week of onset of symptoms. Probably, as the disease stage progresses, those rapid testing protocols will present less sensitivity by the decrease in viral load (Zhao et al., 2020), as well as a higher rate of false negatives due to the low viral load in saliva (Chen J.H. et al., 2020).

For increasing the sensitivity of saliva tests to detect SARS-CoV-2, the instructions should clearly explain the saliva spitting procedure into a container to the individuals, in a “how-to” pamphlet, for example.

### Limitation of the Study

We are aware that our study has limitations as we only have tested a small sample size of individuals, but this is a common problem in studies on emerging infections, making most studies not conclusive. Furthermore, false negatives may occur due to the uncertainty of the first appearance of the symptoms or by technical deficiencies in sampling methodology (Sethuraman et al., 2020).

## Data Availability Statement

The original contributions presented in the study are included in the article/Supplementary Material, further inquiries can be directed to the corresponding author.

## Ethics Statement

The studies involving human participants were reviewed and approved by The Ethical Committee of the Health Secretariat of México, given that the studies were conducted as part of the national COVID-19 surveillance program and were, therefore, part of a routine public health monitoring program conducted by the Mexican government. Written informed consent was not obtained from the individual(s) for the publication of any potentially identifiable images or data included in this article. However, it was explained the right to each individual to decide to participate or not voluntarily. Likewise, before each examination, the present research procedures were explained to the individuals, and a capsule summary of the project and its process were provided. They were informed that the results of the tests would be available if requested.

## Author Contributions

MR-P: conceptualization. MR-P and SRF: methodology, formal analysis, investigation, and writing–review, and editing. LR-M: writing–conceptualization, methodology. MR-P, SRF, LR-M, and ES-M: writing–original draft preparation. BR-B, NF-S, and ES-M: supervision. BR-B: funding acquisition. NF-S: project administration. All authors contributed to the article and approved the submitted version.

## Conflict of Interest

The authors declare that the research was conducted in the absence of any commercial or financial relationships that could be construed as a potential conflict of interest.

## Footnotes

1 https://www.medcalc.org/calc/diagnostic_test.php
